# Abemaciclib in combination with therapies for patients with metastatic breast cancer: a phase 1b study

**DOI:** 10.3389/fonc.2025.1555921

**Published:** 2025-03-12

**Authors:** Sara M. Tolaney, Komal Jhaveri, Teresa Helsten, Shannon L. Puhalla, Alison Conlin, E. Claire Dees, Muralidhar Beeram, Sonya C. Chapman, Andrew Lithio, Lacey M. Litchfield, Matthew P. Goetz

**Affiliations:** ^1^ Department of Medical Oncology, Dana-Farber Cancer Institute, Boston, MA, United States; ^2^ Department of Medicine, Memorial Sloan Kettering Cancer Center, New York, NY, United States; ^3^ Moores Cancer Center, University of California San Diego, San Diego, CA, United States; ^4^ UPMC Hillman Cancer Center, University of Pittsburgh, Pittsburgh, PA, United States; ^5^ Providence Cancer Center, Portland, OR, United States; ^6^ Lineberger Comprehensive Cancer Center, University of North Carolina at Chapel Hill, Chapel Hill, NC, United States; ^7^ South Texas Accelerated Research Therapeutics, San Antonio, TX, United States; ^8^ Eli Lilly and Company, Indianapolis, IN, United States; ^9^ Department of Oncology, Mayo Clinic, Rochester, MN, United States

**Keywords:** abemaciclib, metastatic breast cancer, CDK4, CDK6, everolimus, exemestane, fulvestrant, trastuzumab

## Abstract

**Background:**

The oral, selective, and potent small molecule cyclin-dependent kinases (CDK) 4/6 inhibitor (CDK4/6i) abemaciclib has demonstrated efficacy in advanced breast cancer and high-risk early breast cancer. This Phase 1b study evaluated the safety, tolerability, pharmacokinetics, and antitumor activity of abemaciclib in combination with endocrine therapies (Parts A–D), exemestane + everolimus (Part E), or fulvestrant + LY3023414 (a PI3K/mTOR inhibitor; Part G) in patients with hormone receptor-positive (HR+), human epidermal growth factor receptor 2-negative (HER2-) metastatic breast cancer (MBC), or trastuzumab (Part F), or trastuzumab + pertuzumab (Part H) in patients with HER2-positive (HER2+) MBC.

**Patients and methods:**

This study enrolled women aged ≥18 years old with either HR+, HER2- (Parts E and G), or HER2+ (Parts F and H) MBC. Additional requirements included measurable disease or non-measurable but evaluable bone disease (Parts E and F), or measurable disease (Parts G and H), an Eastern Cooperative Oncology Group performance status of 0–1, and no prior treatment with CDK4/6i (Parts E, F, and H). Adverse events were graded, and tumor response was assessed.

**Results:**

Nineteen patients in Part E received abemaciclib (150 mg, n=15; 200 mg, n=4) with exemestane + everolimus, 24 patients in Part F received abemaciclib (150 mg, n=18; 200 mg, n=6) with trastuzumab, 12 patients in Part G received 150 mg abemaciclib with fulvestrant + LY3023414 (100 mg, n=7; 150 mg, n=5), and four patients in Part H received abemaciclib (100 mg) with trastuzumab + pertuzumab (with prophylactic loperamide). The most common treatment-emergent adverse events (TEAEs) were diarrhea, fatigue, neutropenia, and nausea. Grade ≥3 TEAEs were reported in 16, 18, 10, and 4 patients in Parts E–H, respectively. Abemaciclib had no effect on the pharmacokinetics of the combination study drugs. The objective response rates for patients with measurable disease were 46.2%, 10.0%, 66.7%, and 25.0% in Parts E–H, respectively. A recommended Phase 2 dose was not established for Parts E, G, and H at the dose levels evaluated, and was determined to be 150 mg Q12H in Part F.

**Conclusions:**

Overall, our results demonstrate safety profiles consistent with those previously established for abemaciclib and provide preliminary data for these combination therapies in the treatment of HR+, HER2- or HER2+ MBC.

## 1 Introduction

Breast cancer remains the leading cause of cancer death among women worldwide (1), with most cases diagnosed as hormone receptor-positive (HR+), human epidermal growth factor receptor 2-negative (HER2-) (2). Human epidermal growth factor receptor 2-positive (HER2+) disease, which represents nearly 15% of all breast cancer diagnoses, also remains a therapeutic challenge due to multiple potential mechanisms of therapeutic resistance, despite the availability of anti-HER2 therapies (3–5). Cyclin-dependent kinases (CDK) 4/6 are critical regulators of cell cycle progression by modulating the tumor suppressor retinoblastoma protein (6, 7). CDK4/6 inhibitors (CDK4/6i) have become the standard of care for HR+, HER2- advanced breast cancer (ABC), with efforts underway to extend their benefit through combination therapies and for use in additional populations, including those with HER2+ disease (8, 9).

The oral, selective, and potent CDK4/6i abemaciclib has demonstrated efficacy as a monotherapy or in combination with nonsteroidal aromatase inhibitors (NSAIs) or fulvestrant in the treatment of HR+, HER2- ABC (10–12). Beyond advanced disease, abemaciclib combined with endocrine therapy (ET) also reduced the risk of recurrence in patients with HR+, HER2-, node-positive, high-risk early-stage breast cancer (13, 14).

This study evaluated the safety, tolerability, pharmacokinetics (PK), and antitumor activity of abemaciclib in combination with additional therapies, including exemestane + everolimus or fulvestrant + LY3023414 (a PI3K/mTOR inhibitor) in patients with HR+, HER2- metastatic breast cancer (MBC), or trastuzumab, or trastuzumab + pertuzumab in HER2+ MBC.

## 2 Materials and methods

### 2.1 Study design and objectives

This was a multicenter, nonrandomized, open-label Phase 1b study of abemaciclib in combination with additional therapies for patients with HR+, HER2- (Parts E and G) or HER2+ (Parts F and H) MBC. Results from Parts A–D (ET combination) of this study have been previously published (15). For Parts E–H, patients were enrolled between December 2014 and September 2019.

The primary objective was to evaluate the safety and tolerability of abemaciclib in combination with other therapies, including exemestane + everolimus (Part E), trastuzumab (Part F), fulvestrant + LY3023414 (Part G), and trastuzumab + pertuzumab (Part H). The secondary objectives included evaluation of antitumor activity and PK.

### 2.2 Patients

Women aged ≥18 years diagnosed with either HR+, HER2- (Parts E and G) or HER2+ (Parts F and H) MBC were eligible for the study, depending on the cohort. Patients in Part E (exemestane + everolimus) were required to have received at least one NSAI for metastatic disease and could have been receiving ongoing therapy with exemestane +/- everolimus. Patients in Part G may have received prior treatment with a NSAI for metastatic disease, but this was not required. Patients in Parts F and H were required to have received at least one chemotherapy regimen for metastatic disease and could have been receiving ongoing therapy with trastuzumab (Parts F and H) and/or pertuzumab (Part H only). For Parts E and F, patients were required to have either measurable disease or non-measurable but evaluable bone disease, as defined by the Response Evaluation Criteria in Solid Tumors (RECIST) version 1.1. In Parts G and H, patients were required to have measurable disease as defined by RECIST v1.1. Patients in Parts E, F and H could not have received prior CDK4/6i while patients in Part G were excluded if they received prior therapy with fulvestrant or any PI3K and/or mTOR inhibitor. Regardless of cohort, patients were required to have an Eastern Cooperative Oncology Group performance status (ECOG PS) of ≤1. Additional inclusion criteria for the study have been previously described (15).

### 2.3 Treatment and dosing

Part E consisted of two dose levels of abemaciclib, 150 mg (Cohort 1) or 200 mg (Cohort 2), administered orally every 12 hours (Q12H) on Days 1–28. Patients in Part E also received exemestane 25 mg and everolimus 5 mg once daily on Days 1–28. Planned therapies in Part G included abemaciclib (150 mg Q12H) combined with two dose levels of LY3023414, 150 mg (Cohort 1) or 200 mg (Cohort 2) Q12H, with fulvestrant 500 mg administered via intramuscular injection on Days 1 and 15 of Cycle 1 and Day 1 of Cycle 2 and beyond. Part F included two dose levels of abemaciclib, 150 mg (Cohort 1) or 200 mg (Cohort 2) Q12H, in combination with trastuzumab administered by intravenous (IV) infusion on Day 1 of a 21-day cycle (initial dose of 6–8 mg/kg, followed by subsequent doses of 6 mg/kg). Part H included two planned dose levels of abemaciclib, 100 mg (Cohort 1) or 150 mg (Cohort 2) Q12H, in combination with trastuzumab, as in Part F. Additionally, patients in Part H received IV pertuzumab (initial dose of 420–840 mg, followed by subsequent doses of 420 mg) according to the same schedule as trastuzumab. For patients in Part H, prophylactic loperamide 2 mg was given once daily with the first dose of abemaciclib, and could be discontinued after the first 28 days, per the investigator’s discretion. For Parts E–G, if two or more patients in Cohort 1 experienced dose-limiting toxicities (DLTs), an additional Cohort 0 (Parts E and F: abemaciclib 100 mg Q12H; Part G: LY3023414 100 mg Q12H) could be enrolled.

### 2.4 Dose-limiting toxicities

DLTs were evaluated during Cycle 1 of dose escalation, while DLT-equivalent toxicities (DETs) were evaluated during Cycle 2 and beyond during dose escalation, and Cycle 1 and beyond during dose confirmation. For Parts E-H, the combination maximum tolerated dose (MTD) for abemaciclib was defined as the highest dose of abemaciclib, not exceeding the single-agent MTD (Parts E-G) or combination MTD (Part H), at which fewer than 33% of patients experienced a DLT or DET.

### 2.5 Safety assessments

All patients who received at least one dose of abemaciclib were included in the safety analysis. Adverse events (AEs) were assessed for severity per the National Cancer Institute Common Terminology Criteria for Adverse Events version 4.0 (CTCAE v4.0). Standard laboratory tests were conducted, with relevant hematology and chemistry laboratory values graded per CTCAE v4.0.

### 2.6 Efficacy assessments

This study was not designed to assess efficacy, and all antitumor activity was reported as a secondary objective, with efficacy assessment as previously described (15). In brief, tumor response analyses included the best overall response based on investigator assessment and RECIST v1.1 and were summarized as follows: objective response rate (ORR; complete response [CR] + partial response [PR]), disease control rate (DCR; CR + PR + stable disease [SD]), and clinical benefit rate (CBR; CR + PR + SD ≥24 weeks).

### 2.7 Pharmacokinetics

In parts E, F, and H, PK sampling was performed for all analytes pre-dose on Cycle 1 Day 1, Cycle 1 Day 15, and Cycle 2 Day 1, as well as at 1, 2, 4, 6, 8, and 10 hours after dosing on Cycle 1 Day 1 and Cycle 2 Day 1. In Part H, PK sampling was performed for all analytes pre-dose on Cycle 1 Day 1, Cycle 1 Day 15, and Cycle 2 Day 1, as well as at 1, 2, 4, 6, 8, and 10 hours after dosing on Cycle 1 Day 1 and 2, 4, 6, 8, and 10 hours after dosing on Cycle 2 Day 1. In Parts F and H, additional samples were drawn pre-dose on Day 1 of Cycles 3–5 and after trastuzumab infusion on Day 1 of Cycles 1–5. In part H, additional samples were also drawn after pertuzumab infusion on Day 1 Cycle 4 and 5. In Part G, an additional pre-dose sample was drawn on Cycle 3 Day 1, and fulvestrant samples were obtained pre-dose on Day 1 of Cycles 1–3 and Day 15 of Cycle 1.

Samples were analyzed for abemaciclib (and its metabolites), exemestane, everolimus, fulvestrant, LY3023414, and pertuzumab using a validated liquid chromatography-tandem mass spectrometry method (abemaciclib and metabolites: Q^2^ solutions, Ithaca, NY, USA; exemestane and everolimus: BASi, West Lafayette, IN, USA; fulvestrant: Charles River Laboratories, Montreal, Quebec, Canada; LY3023414: Covance Laboratories Inc, Madison, WI, USA; pertuzumab: Altasciences Company Inc., Quebec, Canada). Trastuzumab was analyzed using a validated Gyrolab xP method (PPD, Richmond, VA, USA).

Standard noncompartmental PK parameters were computed using Phoenix WinNonlin 64 Build 8.0/8.1 (Pharsight Corporation, Mountain View, CA, USA). This analysis included estimation of C_max_, t_max_, C_last_, t_last_, and AUC_0-tlast_. AUC calculations were performed using the log linear trapezoidal method.

### 2.8 Statistical analysis

The study analysis was descriptive only, and no hypothesis testing was planned. Data analyses were performed by study part and dose group. Summary statistics (including number of patients, mean, median, standard deviation, minimum, and maximum) were reported for continuous variables. Categorical data were summarized by the number of patients, frequency, and percentages. Tumor response was analyzed by study part. DLTs were used to determine the sample size for all cohorts. Up to six patients were enrolled per cohort prior to determination of the MTD, with an additional nine to 12 patients potentially enrolled at the MTD (Parts E, F, and G). In Part H, an additional 30 patients could be enrolled after MTD determination.

### 2.9 Ethical approval statement

The study protocol was reviewed and approved by institutional review boards at the participating sites and conducted in accordance with international ethics guidelines, including the Declaration of Helsinki and Council for International Organizations of Medical Sciences International Ethical Guidelines, International Conference on Harmonization Good Clinical Practice guideline, and other applicable laws and regulations. All patients written informed consent prior to participation in the study.

## 3 Results

**Figure 1:**
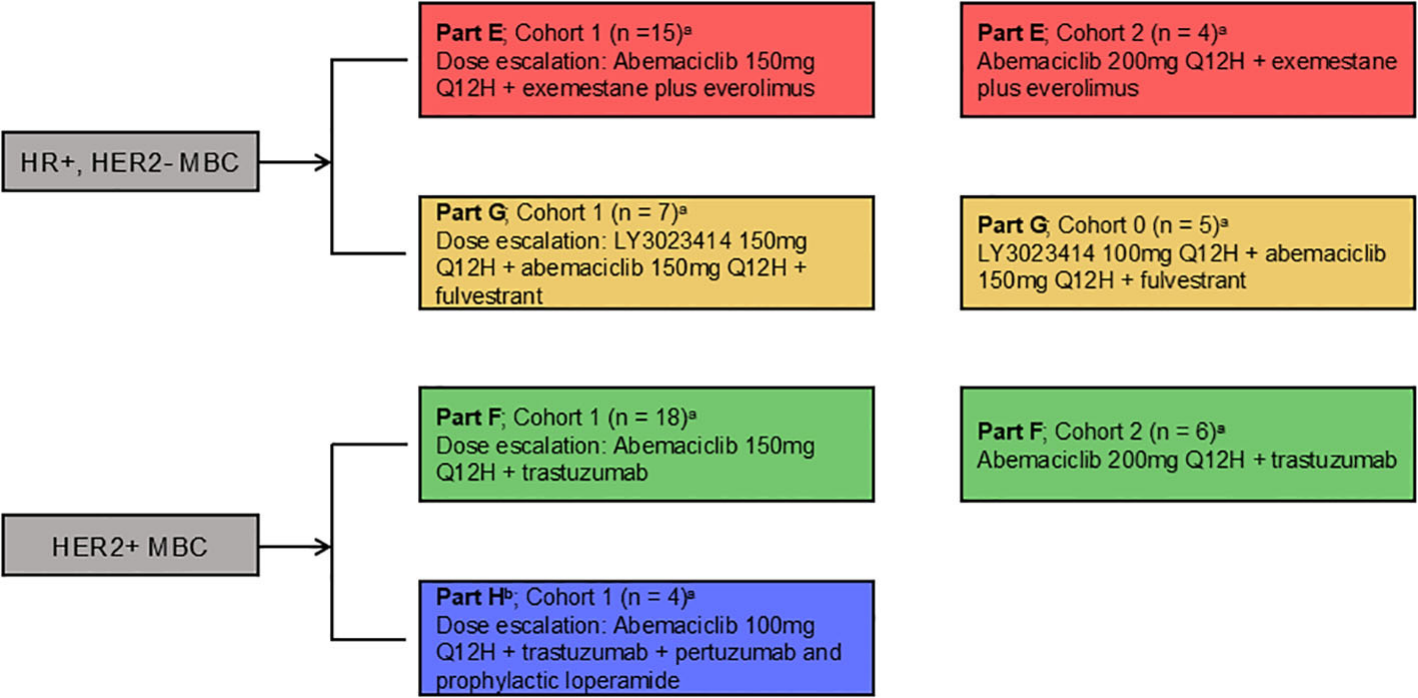
Study design. Women aged ≥18 years diagnosed with HR+, HER2- or HER2+ MBC received abemaciclib orally every 12 hours and the assigned combination therapy. HER2-/HER2+, human epidermal growth factor receptor 2-negative/positive; HR+, hormone receptor positive; MBC, metastatic breast cancer; Q12H, every 12 hours. a: if dose level for Cohort 1 exceeds MTD of the combination MTD, then an additional Cohort 0 (100mg Q12H) could be enrolled b: Part H - cohort 2 not enrolled.

The study design for Parts E–H evaluating abemaciclib in combination with additional therapies is shown in **Figure 1**. Drug dose, number of patients per arm, and cohort are also described.

### 3.1 Part E (abemaciclib plus exemestane and everolimus)

#### 3.1.1 Patients

Nineteen patients were enrolled in Part E (Cohort 1 [abemaciclib 150 mg]: n = 15; Cohort 2 [abemaciclib 200 mg]: n = 4), all of whom received at least one dose of the study treatment (**Table 1**). The median age in Part E was 60 years (range: 41–73 years), including Cohort 1 (57 years) and Cohort 2 (64.5 years). Most patients had an ECOG PS of 1 (n = 11; 57.9%). All patients had received prior systemic therapy, including two patients (10.5%) who had received exemestane and 11 patients (57.9%) who had received exemestane + everolimus.

**Table 1:** Baseline patient and disease characteristics.

	Part E Abemaciclib + Exemestane + Everolimus (N=19)	Part F Abemaciclib + Trastuzumab (N=24)	Part G Abemaciclib + Fulvestrant + LY3023414 (N=12)	Part H Abemaciclib + Trastuzumab + Pertuzumab (N=4)
Age in years, median (range)	60.0 (41.0–73.0)	53.5 (39.0–76.0)	61.0 (39.0–76.0)	66.5 (54.0–72.0)
≥65 years, n (%)	5 (26.3)	4 (16.7)	5 (41.7)	2 (50.0)
Race, n (%)				
White	18 (94.7)	22 (91.7)	12 (100.0)	4 (100.0)
All other	1 (5.3)	2 (8.3)	0 (0.0)	0 (0.0)
ECOG PS, n (%)				
0	7 (36.8)	13 (54.2)	6 (50.0)	3 (75.0)
1	11 (57.9)	11 (45.8)	6 (50.0)	1 (25.0)
2	1 (5.3)	0	0	0
Prior systemic therapies, median (range)^a^	4 (1–6)^b^	8 (3–23)	2 (1–4)	5 (1–10)
HR status				
HR +	19 (100.0)	16 (66.7)	12 (100.0)	2 (50.0)
HR -	0 (0.0)	8 (33.3)	0 (0.0)	2 (50.0)

a Includes chemotherapy, endocrine therapy, adjuvant therapies, neoadjuvant therapies, and metastatic therapies.

b 2 (10.5) and 11 (57.9) patients received prior exemestane or exemestane plus everolimus, respectively.

N, number of patients in the population; n, number of patients in the category; ECOG PS, Eastern Cooperative Oncology Group performance status; HR, hormone receptor.

#### 3.1.2 Dose-limiting toxicities

During dose escalation, one patient each in Cohort 1 (150 mg) and Cohort 2 (200 mg) experienced a DLT of Grade 3 diarrhea. Neither dose exceeded the 33% MTD threshold. Dose confirmation proceeded at the 150 mg abemaciclib dose based on overall experience across Cohorts 1 and 2. The rate of DETs in Cohort 1 was 33.3%, including one patient with Grade 3 pneumonitis, one patient with Grade 3 blood creatinine increased and delirium, one patient with Grade 3 diarrhea, one patient with Grade 3 stomatitis, rash, diarrhea, and hypophosphatemia, and one patient with Grade 4 hyponatremia. The rate of DETs in Cohort 2 was 25.0%, with one patient experiencing Grade 3 pruritus. Considering both the rates of DLT and DET, a MTD for abemaciclib in combination with exemestane + everolimus could not be determined.

#### 3.1.3 Safety

Treatment-emergent adverse events (TEAEs), regardless of causality, observed in Part E are described in **Table 2**. All patients (n = 19) experienced at least one TEAE, and most patients experienced Grade ≥3 TEAEs (n = 16; 84.2%). Overall, the most frequently reported TEAEs included diarrhea, fatigue, neutropenia, nausea, and anemia (**Table 2**). Lung infection (n = 2; 10.5%) was the only serious adverse event (SAE) reported in two or more patients. Five patients (26.3%) experienced SAEs considered by the investigator to be possibly related to study treatment across both Part E cohorts (Cohort 1: 26.7% [n = 4]; Cohort 2: 25.0% [n = 1]).

**Table 2:** Treatment-emergent adverse events, regardless of causality.

TEAE^a^	Part E Abemaciclib + Exemestane + Everolimus (N=19)		Part F Abemaciclib + Trastuzumab (N=24)		Part G Abemaciclib + Fulvestrant + LY3023414 (N=12)		Part H Abemaciclib + Trastuzumab + Pertuzumab (N=4)	
	Any grade n (%)	Grade ≥3 n (%)	Any grade n (%)	Grade ≥3 n (%)	Any grade n (%)	Grade ≥3 n (%)	Any grade n (%)	Grade ≥3 n (%)
Any TEAE	19 (100.0)	16 (84.2)	24 (100.0)	18 (75.0)	12 (100.0)	10 (83.3)	4 (100.0)	4 (100.0)
Diarrhea	17 (89.5)	5 (26.3)	22 (91.7)	8 (33.3)	10 (83.3)	4 (33.3)	3 (75.0)	1 (25.0)
Fatigue	16 (84.2)	1 (5.3)	15 (62.5)	2 (8.3)	8 (66.7)	1 (8.3)	1 (25.0)	0 (0.0)
Neutropenia	13 (68.4)	8 (42.1)	7 (29.2)	4 (16.7)	4 (33.3)	2 (16.7)	1 (25.0)	1 (25.0)
Nausea	12 (63.2)	1 (5.3)	11 (45.8)	0 (0.0)	12 (100.0)	2 (16.7)	0 (0.0)	0 (0.0)
Anemia	11 (57.9)	5 (26.3)	13 (54.2)	3 (12.5)	6 (50.0)	2 (16.7)	2 (50.0)	0 (0.0)
Abdominal pain	10 (52.6)	1 (5.3)	9 (37.5)	1 (4.2)	5 (41.7)	2 (16.7)	1 (25.0)	0 (0.0)
Decreased appetite	10 (52.6)	1 (5.3)	9 (37.5)	1 (4.2)	6 (50.0)	0 (0.0)	1 (25.0)	0 (0.0)
Leukopenia	9 (47.4)	4 (21.1)	5 (20.8)	2 (8.3)	4 (33.3)	4 (33.3)	1 (25.0)	0 (0.0)
Rash	9 (47.4)	1 (5.3)	5 (20.8)	1 (4.2)	5 (41.7)	1 (8.3)	0 (0.0)	0 (0.0)
Vomiting	9 (47.4)	0 (0.0)	8 (33.3)	0 (0.0)	7 (58.3)	0 (0.0)	0 (0.0)	0 (0.0)
Stomatitis	8 (42.1)	2 (10.5)	3 (12.5)	0 (0.0)	4 (33.3)	1 (8.3)	1 (25.0)	0 (0.0)
Cough	7 (36.8)	0 (0.0)	7 (29.2)	0 (0.0)	3 (25.0)	0 (0.0)	0 (0.0)	0 (0.0)
Dyspnea	7 (36.8)	1 (5.3)	5 (20.8)	2 (8.3)	1 (8.3)	0 (0.0)	1 (25.0)	0 (0.0)
Hypokalemia	7 (36.8)	3 (15.8)	6 (25.0)	2 (8.3)	5 (41.7)	3 (25.0)	1 (25.0)	0 (0.0)
Oropharyngeal pain	7 (36.8)	0 (0.0)	3 (12.5)	0 (0.0)	2 (16.7)	0 (0.0)	0 (0.0)	0 (0.0)
Thrombocytopenia	7 (36.8)	2 (10.5)	6 (25.0)	2 (8.3)	2 (16.7)	0 (0.0)	0 (0.0)	0 (0.0)
AST increased	6 (31.6)	1 (5.3)	3 (12.5)	2 (8.3)	1 (8.3)	0 (0.0)	2 (50.0)	2 (50.0)
Dysgeusia	6 (31.6)	0 (0.0)	1 (4.2)	0 (0.0)	1 (8.3)	0 (0.0)	0 (0.0)	0 (0.0)
Alopecia	2 (10.5)	0 (0.0)	3 (12.5)	0 (0.0)	5 (41.7)	0 (0.0)	0 (0.0)	0 (0.0)
Hot flush	1 (5.3)	0 (0.0)	3 (12.5)	0 (0.0)	5 (41.7)	0 (0.0)	0 (0.0)	0 (0.0)
Pruritus	5 26.3)	1 (5.3)	4 (16.7)	0 (0.0)	5 (41.7)	0 (0.0)	0 (0.0)	0 (0.0)
Hypophosphatemia	4 (21.1)	2 (10.5)	1 (4.2)	1 (4.2)	4 (33.3)	3 (25.0)	1 (25.0)	0 (0.0)
Hypotension	0 (0.0)	0 (0.0)	1 (4.2)	0 (0.0)	4 (33.3)	1 (8.3)	0 (0.0)	0 (0.0)
Myalgia	2 (10.5)	0 (0.0)	3 (12.5)	0 (0.0)	4 (33.3)	0 (0.0)	2 (50.0)	0 (0.0)
Blood creatinine increased	4 (21.1)	1 (5.3)	5 (20.8)	2 (8.3)	2 (16.7)	0 (0.0)	3 (75.0)	0 (0.0)
ALT increased	4 (21.1)	1 (5.3)	2 (8.3)	2 (8.3)	1 (8.3)	0 (0.0)	2 (50.0)	1 (25.0)
Dermatitis acneiform	3 (15.8)	1 (5.3)	2 (8.3)	0 (0.0)	1 (8.3)	0 (0.0)	2 (50.0)	0 (0.0)

a Treatment-emergent adverse events that occurred in >30% of patients in at least one part of the study are listed.

N, number of patients in the population; n, number of patients in the category; AST, aspartate aminotransferase; ALT, alanine aminotransferase.

Of the 19 patients in Part E, 15 (78.9%) had at least one abemaciclib dose adjustment, including five patients (26.3%) with at least one dose reduction and 15 patients (78.9%) with at least one dose omission (**Supplementary Table S1**). Diarrhea was the most common AE resulting in dose adjustment.

There were no deaths due to AEs on study treatment or within 30 days of discontinuation from study treatment in Part E reported in the clinical database (**Table 3**). One death due to respiratory failure following pneumonia, not considered by the investigator as potentially related to study treatment, was reported following withdrawal from the study by the patient (not included in **Table 3**). All patients discontinued study treatment, with disease progression being the most frequent cause of treatment discontinuation (n = 8; 42.1%) (**Table 3**). Four patients (21.1%) discontinued treatment due to AEs. No AE resulting in discontinuation occurred in more than one patient.

**Table 3:** Patient disposition.

n (%)	Part E Abemaciclib + Exemestane + Everolimus (N=19)	Part F Abemaciclib + Trastuzumab (N=24)	Part G Abemaciclib + Fulvestrant + LY3023414 (N=12)	Part H Abemaciclib + Trastuzumab + Pertuzumab (N=4)
On treatment	0 (0.0)	0 (0.0)	4 (33.3)	1 (25.0)
Discontinued treatment	19 (100.0)	24 (100.0)	8 (66.7)	3 (75.0)
Reason for treatment discontinuation				
Adverse event	4 (21.1)	1 (4.2)	2 (16.7)	0 (0.0)
Death	0 (0.0)	1 (4.2)	0 (0.0)	0 (0.0)
Non-compliance with study drug	0 (0.0)	0 (0.0)	1 (8.3)	0 (0.0)
Physician decision	5 (26.3)	0 (0.0)	0 (0.0)	0 (0.0)
Progressive disease	8 (42.1)	20 (83.3)	5 (41.7)	3 (75.0)
Withdrawal by patient	2 (10.5)	2 (8.3)	0 (0.0)	0 (0.0)

N, number of patients in the population; n, number of patients in the category.

#### 3.1.4 Efficacy

**Figure 2:**
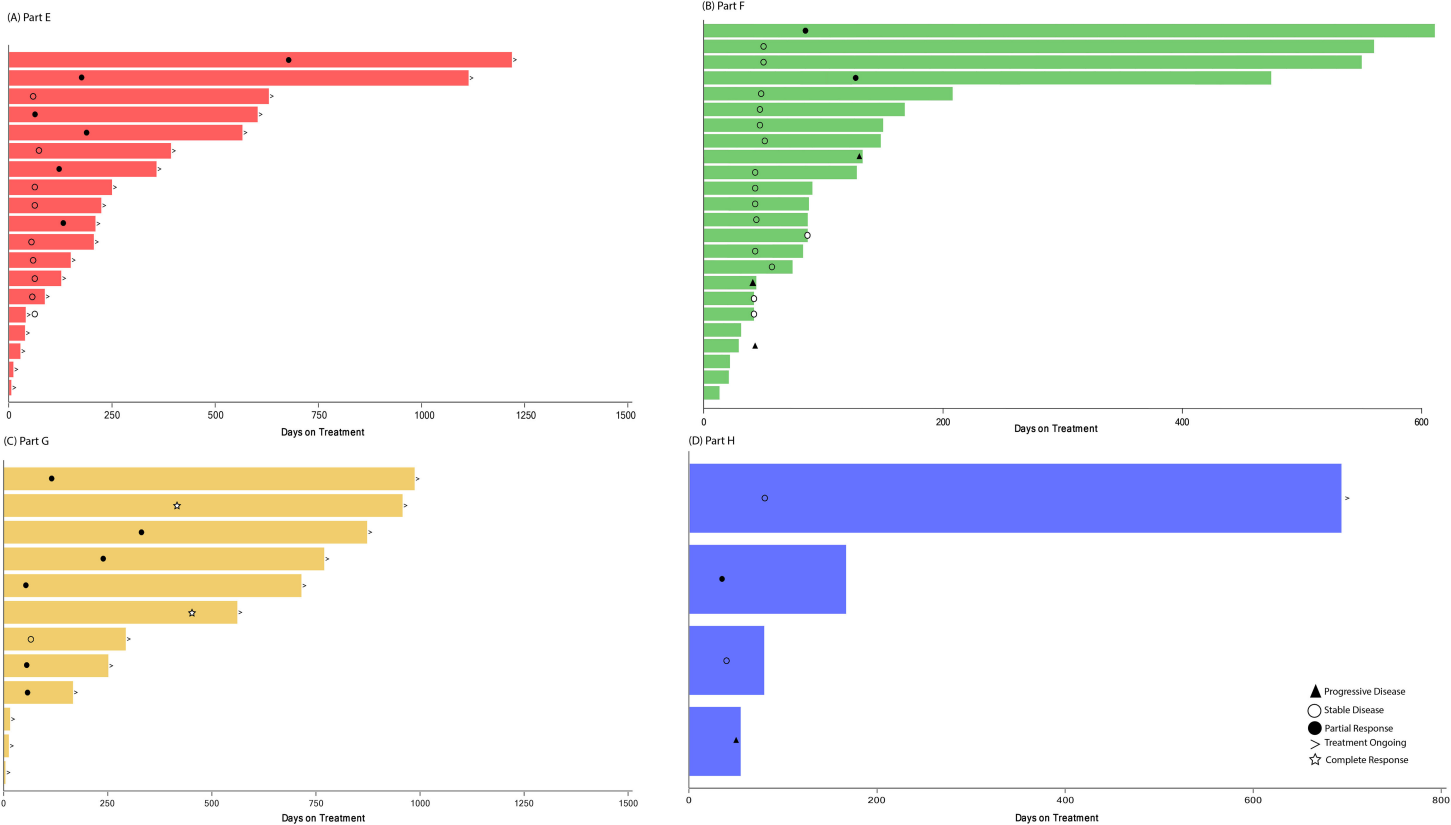
Treatment duration and best overall response. Treatment duration for patients receiving abemaciclib in combination with **(A)** exemestane and everolimus (Part E), **(B)** trastuzumab (Part F), **(C)** fulvestrant plus LY3023414 (Part G), and **(D)** trastuzumab plus pertuzumab (Part H). Best overall response is indicated as: star = complete response; filled circle = partial response; open circle = stable disease; filled triangle = progressive disease; diamond = not evaluable. The > sign indicates treatment ongoing at time of data cutoff.

**Figure 3:**
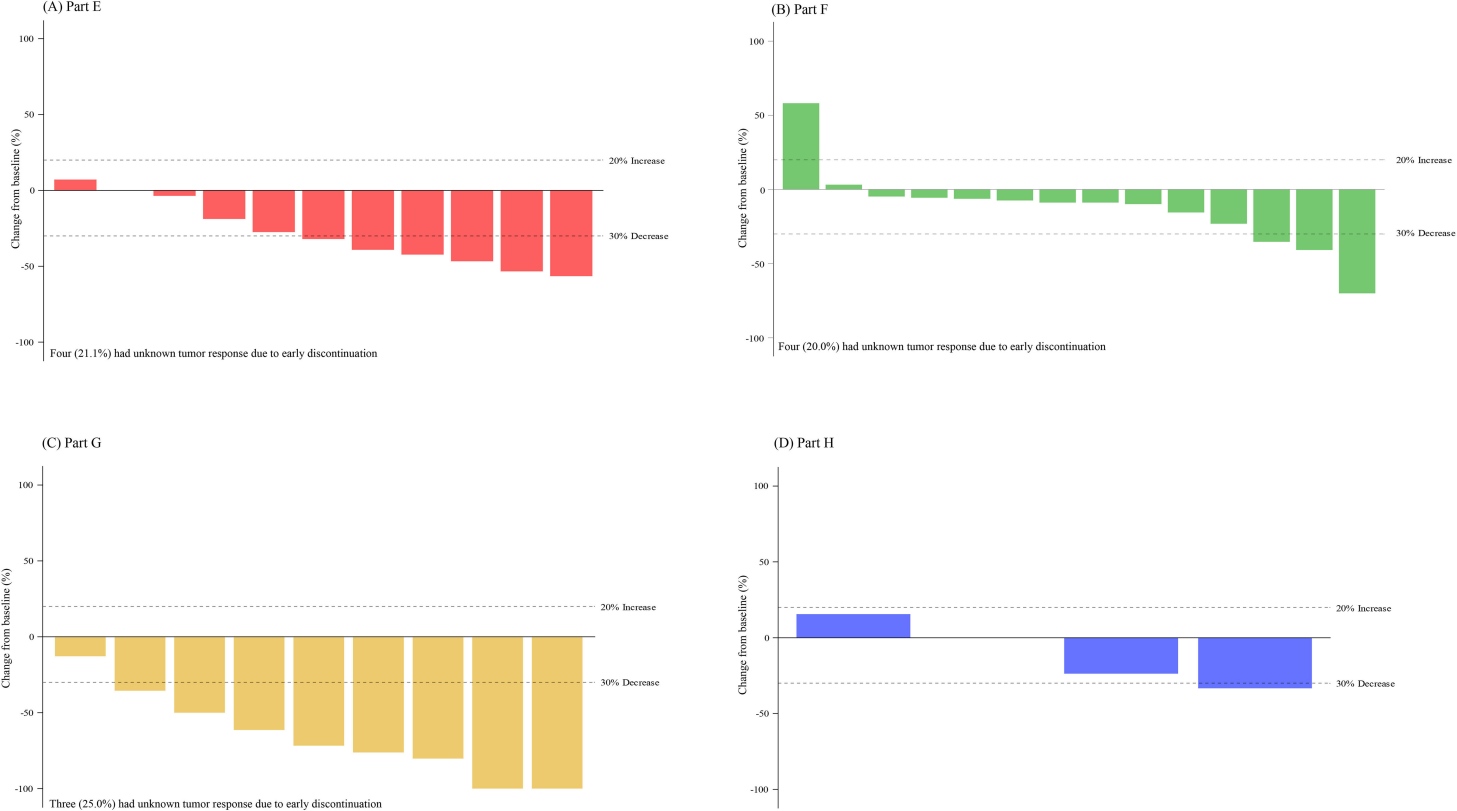
Change in tumor size for patients with measurable disease. Best percent change in tumor size for patients for patients (with measurable disease and available post-baseline assessments) receiving abemaciclib in combination with **(A)** exemestane and everolimus (Part E), **(B)** trastuzumab (Part F), **(C)** fulvestrant plus LY3023414 (Part G), and **(D)** trastuzumab plus pertuzumab (Part H). Change in tumor size greater than 100% is truncated at 100%. Comparison among the study parts is not possible due to differences in patient and disease characteristics and because enrollment opened sequentially.

Treatment duration and best overall response for patients in Part E are displayed in **Figure 2A**. Among the 13 patients with measurable disease, the ORR was 46.2% (0 CR, 6 PR), and DCR was 84.6% (**Table 4**). The results are displayed as a waterfall plot in **Figure 3A**.

**Table 4:** Best overall response in patients with measurable and non-measurable disease by response evaluation criteria in solid tumors version 1.1.

Best overall response^a^	Part E Abemaciclib + Exemestane + Everolimus n (%)	Part F Abemaciclib + Trastuzumab n (%)	Part G Abemaciclib + Fulvestrant + LY3023414 n (%)	Part H Abemaciclib + Trastuzumab + Pertuzumab n (%)
All patients (N)	19	24	12^b^	4^b^
CR	0 (0.0)	0 (0.0)	2 (16.7)	0 (0.0)
PR	6 (31.6)	2 (8.3)	6 (50.0)	1 (25.0)
SD	9 (47.4)	15 (62.5)	1 (8.3)	2 (50.0)
Progressive disease	0 (0.0)	3 (12.5)	0 (0.0)	1 (25.0)
Objective response rate (CR + PR)	6 (31.6)	2 (8.3)	8 (66.7)	1 (25.0)
Disease control rate (CR + PR + SD)	15 (78.9)	17 (70.8)	9 (75.0)	3 (75.0)
Clinical benefit rate (CR + PR + SD ≥24 weeks)	11 (57.9)	5 (20.8)	8 (66.7)	1 (25.0)
Measurable disease (N)	13	20	12	4
CR	0 (0.0)	0 (0.0)	2 (16.7)	0 (0.0)
PR	6 (46.2)	2 (10.0)	6 (50.0)	1 (25.0)
SD	5 (38.5)	12 (60.0)	1 (8.3)	2 (50.0)
Progressive disease	0 (0.0)	2 (10.0)	0 (0.0)	1 (25.0)
Objective response rate (CR + PR)	6 (46.2)	2 (10.0)	8 (66.7)	1 (25.0)
Disease control rate (CR + PR + SD)	11 (84.6)	14 (70.0)	9 (75.0)	3 (75.0)
Clinical benefit rate (CR + PR + SD >24 weeks)	9 (69.2)	5 (25.0)	8 (66.7)	1 (25.0)

a Response according to Response Evaluation Criteria in Solid Tumors version 1.1.

b Patients in Parts G and H were required to have measurable disease.

CI, confidence interval; CR, complete response; N, number of patients in the population; n, number of patients in the category; PR, partial response; SD, stable disease.

#### 3.1.5 Pharmacokinetics

**Figure 4:**
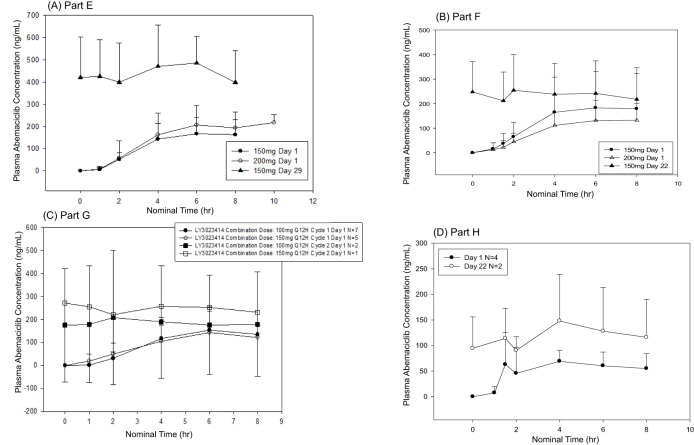
Mean plasma concentrations of abemaciclib following single and multiple doses. Abemaciclib mean plasma concentration versus time profiles for **(A)** Part E (150 mg or 200 mg abemaciclib plus everolimus and exemestane), **(B)** Part F (150 mg or 200 mg abemaciclib plus trastuzumab), **(C)** Part G (150 mg abemaciclib in combination with LY3023414 100 mg or 150 mg and fulvestrant), and **(D)** Part H (100 mg abemaciclib plus pertuzumab and trastuzumab).

Plasma concentration data were available for all 19 patients who received abemaciclib in combination with exemestane + everolimus (n = 15, 150 mg Q12H; n = 4, 200 mg Q12H) (**Figure 4A**, **Supplementary Figure S1A, B**). After a single dose of abemaciclib, the mean C_max_ ranged from 157 ng/mL (Cohort 1, abemaciclib 150 mg, Q12H) to 191 ng/mL (Cohort 2, abemaciclib 200 mg). The single-dose mean AUC_0-tlast_ ranged from 771 hr^*^ng/mL (Cohort 1) to 984 hr^*^ng/mL (Cohort 2). After multiple doses of abemaciclib in Cohort 1 (150 mg Q12H), the mean AUC_0-tlast_ increased to 3280 hr^*^ng/mL, and the mean C_max_ increased to 452 ng/mL, resulting in a mean accumulation ratio of 3.16 (based on C_max_). There were insufficient patient numbers to calculate all mean steady-state PK parameters for patients receiving 200 mg Q12H.

### 3.2 Part F (abemaciclib plus trastuzumab)

#### 3.2.1 Patients

All 24 patients enrolled in Part F (Cohort 1 [abemaciclib 150 mg]: n = 18; Cohort 2 [abemaciclib 200 mg]: n = 6) received at least one dose of study treatment (**Table 1**). The median age of patients in Part F was 53.5 years (range: 39–76 years), which was higher in Cohort 2 (59.5 years) compared to Cohort 1 (52 years). Most patients had an ECOG PS of 0 (n = 13; 54.2%). All patients had received prior systemic therapy (**Table 1**). Sixteen patients (66.7%) had HR+ disease.

#### 3.2.2 Dose-limiting toxicities

No DLTs were reported for patients in Cohort 1 (150 mg) in Part F. In Cohort 2 (200 mg), two patients (33.3%) experienced DLTs of Grade ≥3 diarrhea. Dose confirmation proceeded at the 150 mg abemaciclib dose. At dose confirmation, one patient (5.6%) experienced a DET of Grade ≥3 febrile neutropenia during follow-up (21 days following the last dose of study drug and after starting subsequent therapy). The MTD of abemaciclib in combination with trastuzumab was established as 150 mg Q12H.

#### 3.2.3 Safety

TEAEs, regardless of causality, observed in Part F are described in **Table 2**. All patients (n = 24) experienced at least one TEAE, and most patients experienced Grade ≥3 TEAEs (n = 18; 75.0%). Overall, the most frequently reported TEAEs included diarrhea, fatigue, anemia, nausea, abdominal pain, and decreased appetite (**Table 2**). Two patients (8.3%) experienced study treatment-related SAEs, one patient (4.2%) had febrile neutropenia and the other experienced lung infection (4.2%).

Of the 24 patients in Part F, 19 (79.2%) had at least one abemaciclib dose adjustment, including 11 patients (45.8%) with at least one dose reduction and 18 patients (75.0%) with at least one dose omission (**Supplementary Table S1**). Diarrhea was the most common AE leading to abemaciclib dose adjustment.

There were no deaths due to AEs on study treatment or within 30 days of discontinuation from study treatment. All patients discontinued study treatment, with disease progression being the most frequent reason for treatment discontinuation (n = 20; 83.3%) (**Table 3**). One patient (4.2%) discontinued treatment due to the AE of fatigue.

#### 3.2.4 Efficacy

Treatment duration and best overall response for patients in Part F is shown in **Figure 2B**. Among the 20 patients enrolled in Part F with measurable disease, the ORR was 10.0% (0 CR, 2 PR) and the DCR was 70.0% (**Table 4**). Both patients who experienced a PR had HR+ disease. The results are displayed as a waterfall plot in **Figure 3B**.

#### 3.2.5 Pharmacokinetics

Plasma concentration data were available from 24 patients who received abemaciclib in combination with trastuzumab (n = 18, 150 mg Q12H; n = 6, 200 mg Q12H) (**Figure 4B**, **Supplementary Figure S2A**). Following a single dose of abemaciclib (150 mg or 200 mg), the mean C_max_ values ranged from 133 ng/mL to 148 ng/mL, and the mean AUC0-t_last_ values ranged from 638 hr^*^ng/mL to 713 hr^*^ng/mL. For 150 mg Q12H abemaciclib dosing, the steady-state mean C_max_, AUC0-t_last_, and accumulation ratio based on C_max_ were 242 ng/mL, 1660 hr·ng/mL, and 2.13, respectively. Insufficient patient numbers were available to calculate the mean steady‐state PK parameters for patients receiving 200 mg Q12H.

### 3.3 Part G (abemaciclib plus fulvestrant and LY3023414)

#### 3.3.1 Patients

All patients (n = 12) enrolled in Part G received at least one dose of study treatment (**Table 1**). Patients in Cohort 1 received LY3023414 150 mg (n = 5). Cohort 2 was planned to receive LY3023414 200 mg; however, due to DLTs, this dose was not evaluated, and an additional Cohort 0 (LY3023414 100 mg) was enrolled (n = 7). The median age in Part G was 61 years (range: 39–76 years), which was higher in Cohort 1 (68 years) compared to Cohort 0 (47 years). All patients in Cohort 0 and 4 of the 5 patients in Cohort 1 received prior systemic therapy.

#### 3.3.2 Dose-limiting toxicities

DLTs were reported for two patients (40.0%) in Part G Cohort 1 (LY3023414 150 mg), exceeding the prespecified DLT threshold. Following evaluation of DLTs in Cohort 1, the LY3023414 dose was de-escalated to 100 mg (Cohort 0). At this dose, four patients (57.1%) experienced DLTs, which was also above the prespecified DLT threshold. Therefore, a MTD for abemaciclib in combination with LY3023414 and fulvestrant could not be determined.

DLTs in Cohort 1 included one patient with Grade 3 rash and one patient with Grade 3 dehydration, hypokalemia, gastritis, gastric ulcer, esophagitis, and esophageal ulcer. DLTs in Cohort 0 included two patients with Grade 3 hypophosphatemia, one patient with Grade 3 diarrhea, and one patient with Grade 3 dehydration, hypokalemia, and febrile neutropenia.

As for DETs, one patient in Cohort 1 had Grade 3 stomatitis, and one patient in Cohort 0 had Grade 3 diarrhea.

#### 3.3.3 Safety

TEAEs, regardless of causality, observed in Part G are described in **Table 2**. All patients (n = 12) experienced at least one TEAE, and most patients experienced Grade ≥3 TEAEs (n = 10; 83.3%). Overall, the most frequently reported TEAEs included nausea, diarrhea, fatigue, vomiting, anemia, and decreased appetite. Four patients (33.3%) experienced SAEs considered by the investigator as possibly related to treatment, and no SAE was reported in more than one patient.

Of the 12 patients in Part G, eight (66.7%) had at least one abemaciclib dose adjustment, including six patients (50.0%) with at least one dose reduction and eight patients (66.7%) with at least one dose omission (**Supplementary Table S1**). LY3023414 dose adjustments occurred in 10 patients, including seven with dose reduction and eight with dose omission. Diarrhea was the most common AE resulting in abemaciclib or LY3023414 dose adjustment.

There were no deaths due to AEs while on study treatment or within 30 days of discontinuation from study treatment. Eight patients (66.7%) had discontinued study treatment as of the data cutoff. The most frequent cause for treatment discontinuation was disease progression (n = 5; 41.7%) (**Table 3**). Two patients (16.7%) discontinued study treatment due to AEs, and two additional patients discontinued LY3023414 due to AEs. No AE resulting in discontinuation occurred in more than one patient.

#### 3.3.4 Efficacy

Treatment duration and best overall response for patients in Part G is displayed in **Figure 2C**. Two CR and six PR were observed, demonstrating an ORR of 66.7% and DCR of 75.0% (**Table 4**). The results are displayed as a waterfall plot in **Figure 3C**.

#### 3.3.5 Pharmacokinetics

Plasma concentration data were available for all 12 patients receiving abemaciclib in combination with fulvestrant and LY3023414 (n = 7, 100 mg Q12H; n = 5, 150 mg Q12H) (**Figure 4C** and **Supplementary Figure S1C**). After a single dose of 150 mg abemaciclib with 500 mg fulvestrant and either 100 mg (n = 7) or 150 mg (n = 5) LY3023414, abemaciclib mean C_max_ ranged from 131 ng/mL to 139 ng/mL and abemaciclib mean AUC0-t_last_ ranged from 541 hr^*^ng/mL to 631 hr·ng/mL. Insufficient patient numbers were available to calculate mean steady‐state PK parameters on Cycle 2 Day 1.

### 3.4 Part H (abemaciclib plus trastuzumab plus pertuzumab)

#### 3.4.1 Patients

Part H enrolled four patients in Cohort 1. Dose escalation began with 100 mg abemaciclib Q12H; due to the DLT rate exceeding the prespecified threshold, Cohort 2 was not enrolled. All patients enrolled had received at least one dose of study treatment. The median age was 66.5 years (range: 54–72 years, **Table 1**). Most patients had an ECOG PS of 0 (n = 3; 75.0%). All patients had received prior systemic therapy.

#### 3.4.2 Dose-limiting toxicities

In Part H, three patients (75.0%) experienced DLTs in Cohort 1 (abemaciclib 100 mg), exceeding the maximum prespecified DLT threshold. No additional DLTs or DETs were reported, and further enrollment in this cohort was terminated.

#### 3.4.3 Safety

TEAEs, regardless of causality, observed in Part H are described in **Table 2**. All four patients experienced TEAEs, including Grade ≥3 TEAEs. The most frequently reported TEAEs included diarrhea, increased blood creatinine, alanine aminotransferase (ALT), and aspartate aminotransferase (AST) (**Table 2**). One patient (25.0%) experienced SAEs considered by the investigator as possibly related to study treatment, including Grade 2 abdominal pain, ALT increase, and increased lipase, as well as Grade 3 AST increase.

Three patients (75.0%) required at least one abemaciclib dose adjustment, experiencing at least one dose reduction and omission each (**Supplementary Table S1**).

There were no deaths due to AEs while on study treatment or within 30 days of discontinuation from study treatment. Three patients (75.0%) discontinued study treatment as of the data cutoff due to disease progression (**Table 3**), and no patients discontinued treatment due to AEs.

#### 3.4.4 Efficacy

Treatment duration and best overall response for patients in Part H are displayed in **Figure 2D**. The ORR was 25.0%, including no CR and one PR, and the DCR was 75.0%. Interpretation of these data was limited by the small sample size. The results are displayed as a waterfall plot in **Figure 3D**.

#### 3.4.5 Pharmacokinetics

Plasma concentration data were available for all patients who received abemaciclib in combination with trastuzumab and pertuzumab (**Figure 4D** and **Supplementary Figure S2B, C**). After a single dose of abemaciclib (100 mg), the mean C_max_ was 71.5 ng/mL and the mean AUC_0-tlast_ was 343 hr^*^ng/mL. Mean parameters could not be calculated due to insufficient patient numbers.

## 4 Discussion

Seminal studies have demonstrated the efficacy of abemaciclib as either a single agent (10) or in combination with ET (11, 12, 14) for treating HR+, HEVR2- breast cancer and provided the foundation for the data presented herein. This Phase 1b, multi-part study evaluated the safety and antitumor activity of abemaciclib in combination with additional therapies in patients with HR+, HER2- or HER2+ MBC.

In Part E, patients receiving abemaciclib in combination with exemestane + everolimus for HR+, HER2- MBC showed evidence of clinical benefit, with an ORR of 46.2% for patients with measurable disease. A recommended Phase 2 dose (RP2D) was not established for patients in Part E based on DLTs and DETs at the doses evaluated. The most commonly reported Grade ≥3 TEAEs included diarrhea, neutropenia, anemia, and leukopenia, which is consistent with the previously reported safety profile of abemaciclib (11). Other trials have investigated triplet combinations in HR+, HER2- ABC, such as the TRINITI-1 study which evaluated exemestane, everolimus, and ribociclib after disease progression on prior CDK4/6i. In that study, the safety and efficacy results supported further investigation of CDK4/6 blockade and targeting of the PI3K/AKT/mTOR signaling pathway (16). However, drug-drug interactions were noted, with a lower dose of everolimus being explored in combination with ribociclib.

Part G further investigated the targeting of these pathways, combining abemaciclib and fulvestrant with a PI3K/mTOR inhibitor (LY3023414), demonstrating an ORR of 66.7%. However, DLTs were observed and a RP2D could not be established at the doses evaluated. Similarly, previous studies have shown that the combination of alpelisib or buparlisib with ribociclib and fulvestrant was determined not to be feasible due to toxicity (17). Overall, these results suggest that the addition of a PI3K inhibitor may improve patient benefit compared to CDK4/6i plus ET alone, if toxicity can be managed. The Phase 3 INAVO120 trial evaluated the selective PI3Kα inhibitor inavolisib with fulvestrant and palbociclib as a first-line treatment of phosphatidylinositol-4,5-bisphosphate 3-kinase, catalytic subunit alpha (*PIK3CA*)-mutated, HR+, HER2- ABC, demonstrating a statistically significant improvement in progression-free survival (18).

Preclinical data have implicated cyclin D1/CDK4 in resistance to therapy in HER2+ breast cancer and the potential of CDK4/6i to overcome this resistance (19), providing rationale for the investigation of abemaciclib in combination with HER2-targeted therapy. In Part F, patients were treated with abemaciclib plus trastuzumab. Evidence of clinical benefit was demonstrated, with an ORR of 10% and DCR of 70% in patients with measurable disease. Diarrhea was the most commonly reported AE considered by the investigator to be related to study treatment, regardless of abemaciclib dose. The RP2D was determined to be 150 mg Q12H for this combination and was further evaluated in the Phase 2 monarcHER study, where abemaciclib combined with fulvestrant and trastuzumab significantly improved progression-free survival versus the standard of care chemotherapy plus trastuzumab, while demonstrating a tolerable safety profile (20).

Building on the results from Part F and monarcHER, pertuzumab was added to abemaciclib and trastuzumab in Part H, reflecting that the standard of care first-line treatment for HER2+ breast cancer includes pertuzumab based on the Phase 3 CLEOPATRA trial (21). DLTs restricted the number of patients enrolled in this cohort and limited the interpretation of this regimen’s efficacy. A RP2D was not established for this combination, given the safety profile observed. The ongoing PATINA trial is also evaluating palbociclib in combination with ET and trastuzumab +/- pertuzumab (22).

Throughout this study, PK exposures were evaluated for all combination therapies tested. Abemaciclib exposures were consistent with monotherapy PK data, and no apparent drug-drug interactions were identified for any of the combinations tested.

Overall, the results of this study demonstrated safety profiles generally consistent with those previously established for abemaciclib and provided preliminary data on the anticancer activity of these combinations, contributing to the existing evidence from previously reported Phase 2 and 3 clinical trials of abemaciclib. Given the limited number of patients enrolled, further evaluation in prospective and suitably powered clinical trials is needed to further understand the potential clinical benefit and safety profile of abemaciclib in combination with the additional therapies described herein.

## Data Availability

The raw data supporting the conclusions of this article will be made available by the authors, without undue reservation. Eli Lilly and Company provides access to all individual participant data collected during the trial, after anonymization, with the exception of pharmacokinetic or genetic data. Data are available to request 6 months after the indication studied has been approved in the United States and European Union, and after primary publication acceptance, whichever is later. No expiration date of data requests is currently set once data are made available. Access is provided after a proposal has been approved by an independent review committee identified for this purpose and after receipt of a signed data sharing agreement. Data and documents, including the study protocol, statistical analysis plan, clinical study report, and blank or annotated case report forms will be provided in a secure data sharing environment. For details on submitting a request, see the instructions provided at www.vivli.org.
