# Supplementary material for: Abemaciclib in combination with therapies for patients with metastatic breast cancer: a phase 1b study
Source: Front Oncol. 2025 Mar 12;15:1555921. doi: 10.3389/fonc.2025.1555921 (PMC11937066; doi:10.3389/fonc.2025.1555921)

**Supplementary Information**

**Abemaciclib in Combination with Therapies for Patients with Metastatic Breast Cancer: A Phase 1b Study**

Sara M. Tolaney,^1*^ Komal Jhaveri,^2^ Teresa Helsten,^3^ Shannon L. Puhalla,^4^ Alison Conlin,^5^ E. Claire Dees,^6^ Muralidhar Beeram,^7^ Sonya C. Chapman,^8^ Andrew Lithio,^8^ Lacey M. Litchfield,^8^ Matthew P. Goetz^9^

^1^Dana-Farber Cancer Institute, Boston, MA, USA

^2^Memorial Sloan Kettering Cancer Center, New York, NY, USA

^3^University of California San Diego, San Diego, CA, USA

^4^University of Pittsburgh, Pittsburgh, PA, USA

^5^Providence Cancer Center, Portland, OR, USA

^6^University of North Carolina at Chapel Hill, Chapel Hill, NC, USA

^7^South Texas Accelerated Research Therapeutics, San Antonio, TX, USA

^8^Eli Lilly and Company, Indianapolis, IN, USA

^9^Mayo Clinic, Rochester, MN, USA

*** Corresponding author:**Sara_Tolaney@dfci.harvard.edu

**Supplementary Table 1:** Dose adjustments and omissions for abemaciclib

| n (%) | Part E  Abemaciclib + Exemestane +  Everolimus  (N=19) | Part F  Abemaciclib + Trastuzumab  (N=24) | Part G  Abemaciclib + Fulvestrant + LY3023414  (N=12) | Part H  Abemaciclib + Trastuzumab +  Pertuzumab  (N=4) |
| --- | --- | --- | --- | --- |
| Patients with >1 dose adjustment or omission | 15 (78.9) | 19 (79.2) | 8 (66.7) | 3 (75.0) |
| Patients with dose reduction | 5 (26.3) | 11 (45.8) | 6 (50.0) | 3 (75.0) |
| Patients with dose omissions | 15 (78.9) | 18 (75.0) | 8 (66.7) | 3 (75.0) |
|  | | | | |

**Supplementary Figures**

**Supplementary Figure 1:** Mean plasma concentration-time profiles of **(A)** exemestane and **(B)** everolimus in Part E and **(C)** LY3023414 (100 mg or 150 mg Q12H) administered in combination with abemaciclib 150 mg Q12H and fulvestrant in Part G


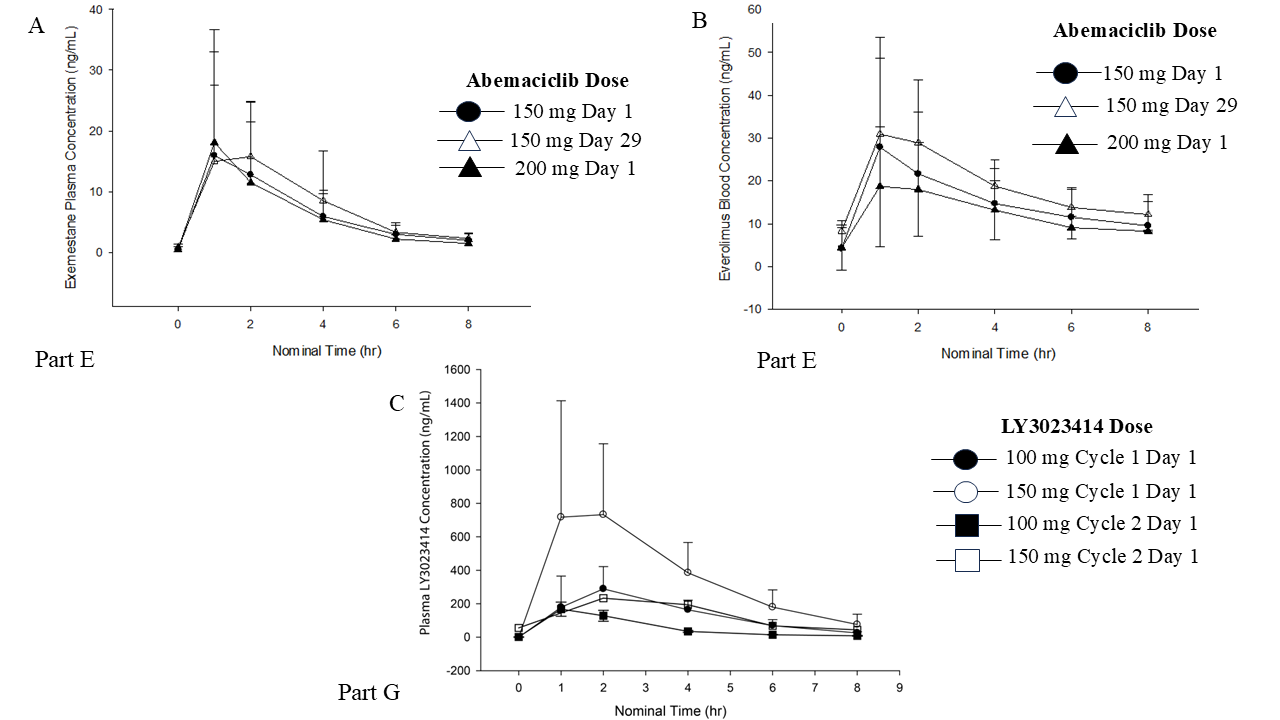


**Supplementary Figure 2:** (A) Part F -Mean plasma concentration-time profiles of trastuzumab. (B) Part H - Pertuzumab mean serum concentration versus time profiles after a single trastuzumab dose of 840 mg on Day 1 and 420 mg on Day 22 in combination with trastuzumab and abemaciclib. (C) Part H -Trastuzumab mean serum concentration versus time profiles after a single trastuzumab dose of 8 mg/kg on Day 1 and 6 mg/kg on Day 22 in combination with pertuzumab and abemaciclib.


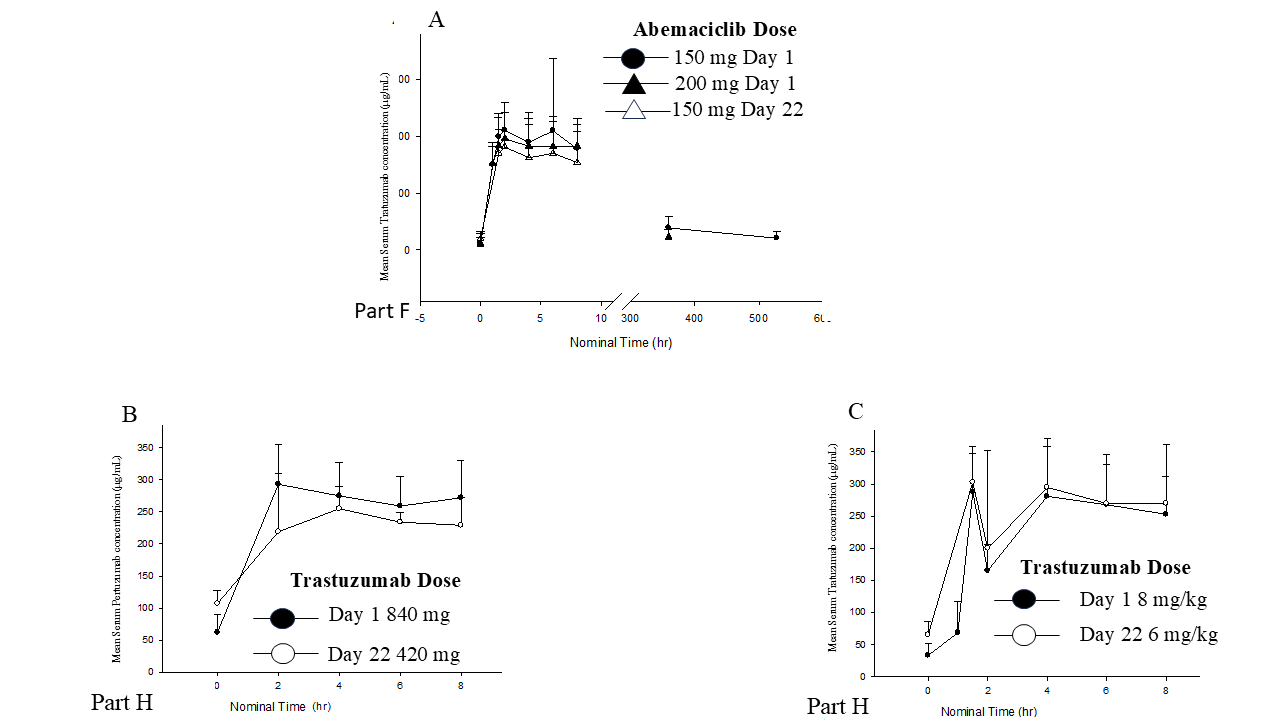

Supplement: Supplementary file 1 [file DataSheet1.docx]
